# Influence of Orthodontic Rapid Maxillary Expansion on Nocturnal Enuresis in Children

**DOI:** 10.1155/2015/201039

**Published:** 2015-08-16

**Authors:** Lidia Hyla-Klekot, Marek Truszel, Andrzej Paradysz, Lidia Postek-Stefańska, Marcin Życzkowski

**Affiliations:** ^1^Department of Pediatric Nephrology, Pediatrics and Oncology Center, 41-500 Chorzów, Poland; ^2^Private Dentistry Practice, 43-300 Bielsko-Biała, Poland; ^3^Department and Clinic of Urology, Medical University of Silesia, 41-800 Zabrze, Poland; ^4^Department and Clinic of Pediatric Dentistry, Medical University of Silesia, 41-800 Zabrze, Poland

## Abstract

*Background*. The etiology of nocturnal enuresis (NE) is multifactorial and has not been fully explained yet. New ways of treatment are constantly being investigated, including the rapid maxillary expansion (RME). *Methods*. A total of 41 patients diagnosed with NE were divided into two experimental groups: A and B. Group A included 16 children who have been treated with RME. Group B comprised 25 children who have not undertaken orthodontic treatment. Children from both groups have been monitored in monthly intervals, during a 12-month period, towards the intensification of NE. The comparative analysis of both groups has been conducted after 3 years of observation. *Results*. Statistical analysis has shown a 4.5 times increase of the probability of reduction of NE in the case of the treated group in comparison with the group of children who have not undergone orthodontic treatment. Unfortunately, the chance of obtaining total dryness diminished proportionally to the higher degree of intensification of enuresis at the beginning of the test. *Conclusion*. RME can constitute an alternative method of NE treatment in children, irrespective of the occurrence of upper jaw narrowing.

## 1. Introduction

Nocturnal enuresis (NE) in children is one of the most complex psychosomatic phenomena which takes place in the period of human development and maturation. Its multiple factor conditioning and not fully defined etiology make the problem the subject of interest for many doctors of various specialties: pediatricians, urologists, psychiatrists, nephrologists, and child neurologists as well as psychologists. The results of treating NE deviate from its spontaneous relief to total lack of reaction to all available methods of treatment. In connection with the fact that it is a major scale problem, new ways of treatment are constantly searched for. In the literature one can find sparse information about the positive influence of rapid maxillary expansion (RME) on the results of treating NE even in those children who were never diagnosed with its narrowing [1–5].

NE is conditioned by multiple factors. The genetic influence, autosomal dominant inheritance with penetration (expression) exceeding 90%, environmental conditions (social factors, parents' expectations, and toilet training), somatic conditions (nocturnal polyuria, decreased nocturnal functional bladder capacity, narrowing of upper respiratory tract (URT), sleeping disorders, and delayed maturation of central nervous system), and psychological conditions (lack of interest from parents or guardians or the fact of staying in nursing homes, stress) are taken into account [6, 7].

Disregarding the genetic impacts, it is now considered that there are two main pathogenetic mechanisms of NE which are sufficiently scientifically documented: nocturnal polyuria and high threshold of night-time awakenings [8, 9].

Single reports in literature about the potential connection of bed-wetting with the anatomical conditions of URT as well as upper jaw dimensions became the inspiration to proceeding with the examinations herein which would allow for obtaining the answer to the following questions: (1) Is there a connection between the occurrence of NE and morphology of the chewing organ in children, including, especially, the size of upper jaw? (2) Does orthodontic treatment involving RME influence the level of bed-wetting intensity?

## 2. Material and Methods

41 patients with diagnosed NE (24 boys and 17 girls) referred by doctors from the pediatric nephrology department of Chorzow Centre of Pediatrics and Oncology participated in general in the initial examination. The inclusion criteria for treatment through RME were as follows: age of 6–18 years, present NE, correct secretion function of kidneys, lack of infection of urinary tract as well as URT, consent for participation in the research given by the child's legal guardians, filling in of the initial information survey, and replying to final questions concerning NE after 3 years. The exclusion criteria for RME involved not meeting any of the inclusion criteria, active teeth caries which did not allow for carrying out orthodontic treatment, poor oral cavity hygiene, insufficient number of teeth for fixing the appliance, and lack of sufficient motivation to cooperation during orthodontic treatment.

Their parents or legal guardians filled in the initial information survey completely and replied to the final questions, which concerned the occurrence and frequency of bed-wetting after 3 years of observation. The initial survey contained questions about disorders present in children related to URT (tonsils enlargement, allergy, and frequent infections), sleeping with open mouth, sleeping character (calm or not), snoring, grinding of the teeth, sweating at night, morning tiredness, waking up of a child after bed-wetting, results obtained at school, parents' divorce, bed-wetting in the family history, child's emotional condition (impatient, lively, sleepy, calm, etc.), bed-wetting characteristic features (whether the child wets the bed from the day it was born without intervals, bed-wetting intensity level, and treatment methods applied and their effects). Intensity level of NE was described with the use of a 4-grade scale prepared for the purposes of the examination, that is, 4: a couple of times during the night, 3: once during the night, 2: once or a couple of times during the week, and 1: once or a couple of times during the month.

The orthodontic part involved preparing and analysis of diagnostic dental casts. The analysis of dental casts was performed in comparison to generally assumed standards for white Caucasian race. Due to high percentage of malocclusion in general population, it was decided not to compare the results in children with diagnosed NE and healthy children, who do not wet the bed.

The children were divided into two research groups: A and B. Due to multiple-reason and complex character of NE, it was not planned to compare the results obtained with the control arm. 16 children (9 boys and 7 girls) with diagnosed NE were qualified to research group A, whose parents or guardians accepted RME treatment. Research group B was composed of 25 children (15 boys and 10 girls) whose parents or guardians did not express such consent or met the exclusion criteria. Children from both groups A and B were monitored in monthly intervals for the period of 12 months towards the intensification of bed-wetting—possible reduction of bed-wetting frequency in the accepted 4-grade scale or achieving total dryness. Additionally, in children from group A, for the purposes of documenting the effects of RME on the occlusion, control diagnostic models were made after taking the appliance off and they were subjected to the same analysis as at the beginning of research. After 3 years of observation, at the end of research, parents and guardians of children from both groups A and B were asked to reply to the question concerning the occurrence and frequency of bed-wetting in order to compare the influence of various methods of treatment applied; in case of children from group A it involved also RME, on NE intensity level. The replies obtained were compared with the replies from the beginning of the research and assessed in the following sequence: (1) bed-wetting reduction in grades (with reference to created 4-grade bed-wetting scale), (2) bed-wetting reduction (in general), and (3) complete cessation of bed-wetting. The examination protocol was approved by the Bioethical Commission of the Medical University of Silesia, decision number NN-6501-87/06. The trial has been registered and allocated as ACTRN12614000899640.

In order to explain what consequences may RME have with regard to the results of NE treatment, the assumptions of logistic regression were applied. For the purposes of assessing the influence of RME on the treatment results, that is, achieving defined level of bed-wetting reduction, the ordinal scale was assumed of applicable reduction levels and it was based on the assumptions of ordinal logistic regression. The results of regression parameters achieved were interpreted with the use of odds ratio.

## 3. Results

**Figure 1:**
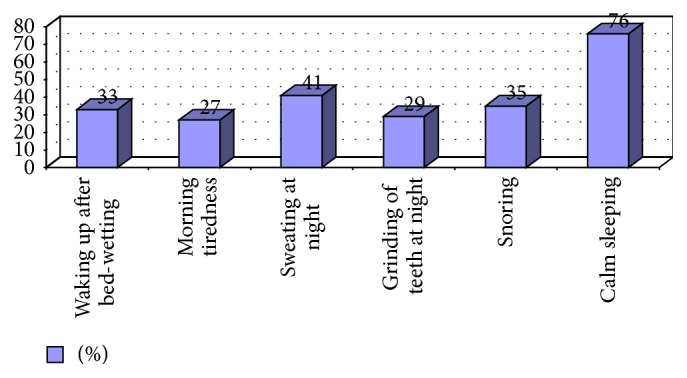
Characteristics of the sleep.

Based on the survey, the frequency of the given disorders in terms of URT was determined, while in sleeping almost 50% of the patients' lips remained open. Enlargement of tonsils, allergy, and chronic infections of URT were present on average in 10% of examined people. The majority of children in their guardians' opinion slept calmly (76%) and deep enough that, after wetting the bed, 66% kept sleeping. 25% of the children would wake up tired; 33% were snoring or grinding teeth. 41% of children sweated excessively while sleeping (Figure 1). In 43% of children, NE was present also in one or more persons in the family. A considerable group of children (29%) in the parents' opinion did not achieve good results at school. In 14% of cases, children's parents were divorced. In reply to the question regarding the emotional condition of examined children, parents considered them as impatient (39% of replies) and sleepy (43%) as well as lively (63%) and calm (72%). In most cases, parents specified more than one character feature of their children sometimes joining the contradictory ones. Most children wetted the bed since the day they were born without intervals (34 children, 67%). Most often they did it once during the night (16 children, 31%) or at least once during the week (16 children, 31%), more rarely a couple of times during the night (9 children, 18%). Sporadic bed-wetting or a couple of times during the month was reported by 10 respondents (20%).

The most frequent way of NE treatment was administration of drugs. Liquid control was rarely applied (8%), as well as waking up and visiting a psychologist (12% each) and other methods, including acupuncture and bioenergy healing (20%). In 14% of cases no treatment was applied. Hitherto, therapy in the group of children subjected to survey examination appeared to be successful (bed-wetting frequency in children was reduced) in 43%; however, the problem did not subside in total, while in 20% of children it returned with time on the same level.

In orthodontic examination, it was determined that the most frequent malocclusion is class II (35%) quite often complicated with deep bite (33%). Lateral crossbite was recorded in 14% of the children examined. The rarest were class III (4%) and open bite (2%).

**Figure 2:**
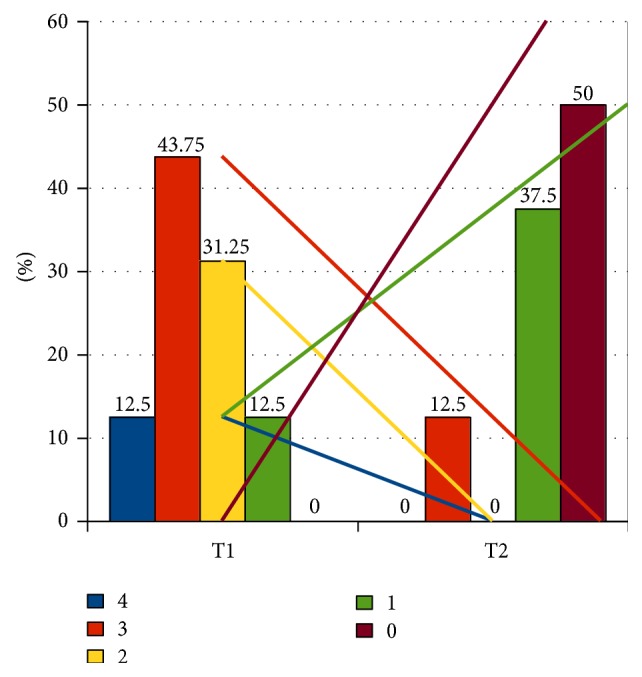
Bed-wetting intensity in grades in group A. T1: at the beginning of examination. T2: at the end of examination after 3 years. 4, 3, 2, 1, and 0: reduction of NE in the number of grades with line regression for each grade.

**Figure 3:**
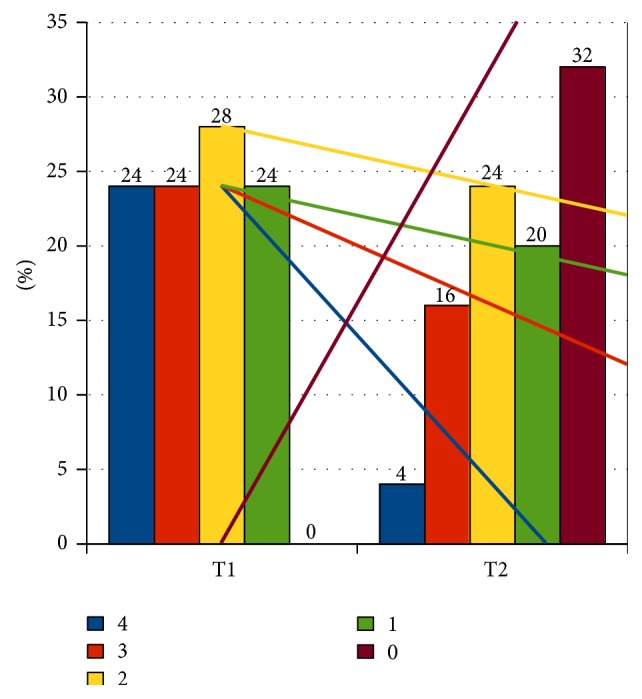
Bed-wetting intensity in grades in group B. T1: at the beginning of examination. T2: at the end of examination after 3 years. 4, 3, 2, 1, and 0: reduction of NE in the number of grades with line regression for each grade.

**Figure 4:**
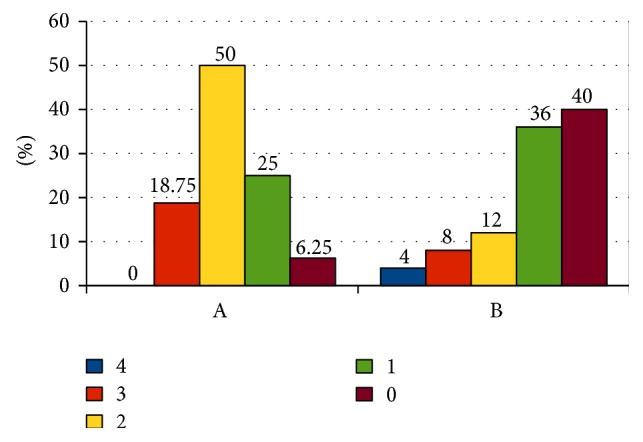
The number of grades by which the bed-wetting intensity decreased within both groups. A: group A. B: group B. 4, 3, 2, 1, and 0: reduction of NE in the number of grades.

In the examined group A under the influence of RME average upper jaw widening obtained was on the level of 6.5 mm (min. 4.6–max. 8.2). The frequency of bed-wetting in children in both examined groups and decrease of its intensity during the observation period are presented in Tables 1 and 2 as well as Figures 2 to 4. Right after widening (in the period of 3 months from fixing the appliance, so after achieving the planned goal) 10 out of 16 children in group A did not wet the bed at all and the effect was stable in the longer observation period (8 out of 16 children were dry after 36 months). In case of 5 children, the frequency of bed-wetting decreased with one or two grades, while in one patient no improvement was noted—he kept wetting the bed every night. Two children, who right after the widening did not wet the bed, after 6 and 10 months, respectively, started bed-wetting again but it took place occasionally—once a month.

As it was presented on Figures 2 and 3, the percentage of occurrence of the given grades in the 4-grade scale of bed-wetting and direction and angle of inclination of regression curves (separately for each level) state the greater intensity of bed-wetting reduction in group A in comparison to group B. If the number of grades which the reduction of bed-wetting intensity took place with is taken into account, then, in case of group A, a greater percentage of children achieved the decrease with at least 2 grades or more (68.75%) in comparison to children from group B (24%) (Figure 4). In group A, bed-wetting decreased with at least one grade in almost all children (with the exception of one), while in group B the same effect was noticed only in 15 out of 25 children (applicable percentages: 93.75% in group A and 60% in group B). After 3 years of observation, half of the children in group A did not wet the bed at all, while in group B only 32% of the children examined did not wet the bed.

In the comparative analysis of examined groups A and B first data from the survey carried out at the beginning of the research was taken into account. 41 patients were analyzed in total (16 from group A and 25 from group B) based on 22 variables: (1) group of children A or B, (2) age, (3) infections of URT, (4) allergies, (5) enlarged tonsils, (6) mouth open while sleeping, (7) calm sleeping, (8) snoring, (9) grinding of teeth at night, (10) sweating at night, (11) morning tiredness, (12) waking up after wetting the bed, (13) good results at school, (14) divorced parents, (15) NE in the family history, (16) emotional condition, (17) bed-wetting frequency, (18) bed-wetting since the day they were born without intervals, (19) medicines taken, (20) liquid control, (21) waking up, and (22) consultations with a psychologist and they were compared with 3 final variables, obtained after 3 years of observations: (a) bed-wetting reduction in 4-grade scale, (b) bed-wetting reduction (in general), and (c) complete cessation of bed-wetting.

From the selected risk factors, with the use of statistic tools, logistic regression and ordinal logistic regression, the variables were selected characterized with statistically essential influence on occurrence of NE episodes in children. The calculations were carried out with the use of both classic and Bayesian techniques.

The probability of increase in the reduction level, that is, bed-wetting decrease with 1 level (grade), increased 4.5 times as far as group A is concerned (orthodontically treated) in comparison to group B, children not subjected to treatment by means of RME. No statistically essential impact of any of the factors listed in the research survey on the reduction of NE was observed. The probability of bed-wetting reduction in general increased 10 times in case of group A in comparison to group B. The results are confirmed by the estimated value of odds ratio obtained with the use of logistic regression by means of Bayesian method; in this case, the chance of reducing NE was estimated for the value 7.5 times higher in children subjected to orthodontic treatment, and so, more or less, 50% decrease of the probability of total cessation of bed-wetting in case of intensity 1 degree higher at the beginning of the research was achieved. At the same time, in patients who differed with 2 degrees in the assumed bed-wetting scale the chance of achieving total dryness decreases with ca. 75%.

## 4. Discussion

In the world literature, one can find recommendations of many various methods of treating NE. It only certifies that, until today, the one method, successful for most children, has not been found yet. One shall also not expect to find it in the future due to very complex and multiple factor etiology of the disorder. In the recent years, however, some codes of conduct, both in terms of diagnosis and therapy, were worked out. The children who wet the bed shall undergo laryngological examination in terms of upper airway obstruction (UAO) and occurrence of obstructive sleep apnea (OSA). Some authors consider NE as one of the symptoms of OSA, and also it may portend the occurrence of OSA symptoms in the future [10–14]. It is also worth noticing that bed-wetting relieves after successfully completing treatment of OSA [12, 15–17].

The improvement of air flow in the URT decreases the intensity of NE decisively. From physiological point of view, nasal cavity is responsible for ca. 50% of breath resistance and its anatomical or functional obstruction is also an essential factor of OSA risk [18]. So, the reduction of breath resistance in the nasal cavity by means of RME seems to be an efficient method for treating OSA and there are many reports about it in literature. The best results may be achieved if a child with the diagnosed OSA has a narrowed maxilla but not enlarged tonsils [19]. Connecting OSA with NE provoked the orthodontists to deal with the subject as the narrowing of maxilla which makes up the bottom of nasal cavity may be one of the reasons of UAO. It has to be underlined that orthodontic treatment will not be efficient in treating NE in patients for whom nocturnal polyuria or decreased nocturnal functional bladder capacity is the reason of the problem. RME may have influence only in cases in which the main reason is difficult waking up of a child from sleeping after complete filling up of the bladder.

The positive side effect consequence of RME is widening of the upper jaw skeletal structure which additionally increases both the section and capacity of the nasal cavity. Thanks to it, RME may restore the nasal breathing tract even in 92.8% of cases to children who habitually breathe through the mouth [20, 21]. Besides, RME thanks to widened palate and frequently present further orthodontic treatment after RME and harmonization of dental arches may indirectly improve anatomic relationships in the oropharyngeal space by means of modification of the tongue resting position [22, 23].

The positive result of RME is not necessarily conditioned by initial presence of upper jaw obstruction which manifests with lateral crossbite or at least lack of place for teeth, only the fact of upper jaw widening and change of morphological conditions in URT. In the research herein, the percentage of children with the crossbite diagnosed in the examined group A amounted to 31% (5 children out of 16). In general, only in 18 children (from the total number of 41, what gives the percent of 44%) were diagnosed with unilateral or bilateral crossbite. The reduction of children bed-wetting frequency achieved in all examinations was not connected with the presence of crossbite, which is characteristic for upper jaw narrowing. A conclusion may be drawn then that presence of crossbite is not a good parameter in order to qualify the patient for NE treatment through RME.

In the examination herein, among 5 children in group A, in which crossbite was diagnosed at the beginning of research, improvement was recorded in all, while total dryness was achieved only in two children (40%).

The process of orthodontic treatment directed to RME and change of the chewing organ morphology in children caused essential improvement in terms of decreasing the frequency of episodes of NE or cessation of symptoms that is achieving total dryness in long-term observation. What is more, the result obtained was stable in the 3-year observation period and does not involve side effects. Similar remarks were made also by other authors [1–4].

NE treatment by means of RME shall surely not be recommended as a first-line therapy; behavioral therapy and alarm shall be considered as such. When assessing the hitherto applied methods of treating bed-wetting in the work, which often were unsuccessful, medicines application—mostly desmopressin—and waking alarm were dominant. The fact of relatively rarely applied treatment with the use of an alarm among Polish children shall be noticed. Additionally, due to limitation of application of desmopressin on the area of the European Union, the second-line therapy in children with enlarged tonsils may involve its removal, while if these are not present or when surgical treatment does not lead to desired results, one shall consider orthodontic treatment. RME does not then aim at correcting the malocclusion but only at reducing NE. RME is a rather rapid and noninvasive method and in case of good oral cavity hygiene does not lead to any side effects and complications.

To sum up the discussion, it shall be noticed that bed-wetting is more often present in patients with URT obstruction and breathing disorders while sleeping and also in those with high threshold of waking up from sleeping (the so-called* deep sleepers*) [10–14, 16, 24–28]. On the other hand, in a lot of research, the advantageous impact of RME on the results of treating respiratory disorders while sleeping was proven [19, 20, 29–32]. RME has the same, beneficial impact also on reduction of NE, what the results achieved not only in the research but also in other authors' research speak for [1–4, 33]. In the research herein it was planned to expand the upper jaw only in transverse dimension. Bearing in mind the relationship between the greater values of Popovich index achieved, what certifies of upper jaw shortening, and greater resistance to NE frequency reduction as a consequence of only RME which enlarges the maxilla only in transverse dimension, it may be worth proceeding with wider examination involving the upper jaw increase also in sagittal dimension with the use of, for example, a facial mask. Considering the report on advantageous impact of lower jaw protraction on bed-wetting reduction, in the author's opinion it would be worth to examine the impact of such appliances in a larger group of examined people.

After statistical analysis no relationships between psychological factors such as stress, school results, parents' divorce, and intensification of NE were found. Despite the fact that some authors notice such connection, similar results were obtained by other researchers and further testing on it is not recommended [2–4, 34–39]. Because of the most probable mechanism of action of RME impact on NE, the inclusion of polysomnographic testing (related to testing blood saturation with respiratory gases and sleeping quality examination) to a set of examinations carried out on children with resistant to treatment bed-wetting seems justified mostly in order to exclude the presence of OSA, as the reason of disorder. Untreated OSA at developmental age may have much more essential health consequences in the course of further patient's life than even the most persistent NE, which is not direct threat to health and life.

## 5. Conclusions

The results of research obtained allow for formulation of the following conclusions:

1. The morphology of upper jaw is not connected with the occurrence of nocturnal enuresis and cannot be the agent which qualifies to successful treatment of bed-wetting through rapid maxillary expansion.
2. Orthodontic treatment which involves rapid maxillary expansion may be an alternative way of treating nocturnal enuresis in children, regardless of upper jaw narrowing presence.
3. Physiological mechanism of action responsible for the positive result of orthodontic treatment remains unexplained and requires further research. It seems justified to include polysomnographic testing to the set of examinations on children with resistant to treatment bed-wetting in order to exclude the occurrence of obstructive sleep apnea as the reason of disorder.

## Figures and Tables

**Table 1:** Bed-wetting frequency in children in the examined group.

Examined group	Frequency of bed-wetting									
	T1					T2				
	4	3	2	1	0	4	3	2	1	0
A (*n* = 16)										
*n*	2	7	5	2	0	0	2	0	6	8
%	12.5	43.75	31.25	12.5	0	0	12.5	0	37.5	50
B (*n* = 25)										
*n*	6	6	7	6	0	1	4	6	5	8
%	24	24	28	24	0	4	16	24	20	32

*n*: the number of children in the group with the given bed-wetting level.

T1: at the beginning of the research.

T2: at the end of the research after 3 years.

**Table 2:** Reduction of NE in the examined groups at the end of examination after 3 years of observation.

Examined group	Reduction (in the number of grades)					Reduction (in general)	Total dryness
	4	3	2	1	0		
A (*n* = 16)							
*n*	0	3	8	4	1	15	8
%	0	18.75	50	25	6.25	93.75	50
B (*n* = 25)							
*n*	1	2	3	9	10	15	8
%	4	8	12	36	40	60	32

*n*: the number of children in the group with the given bed-wetting intensity.

## References

[1] Timms D. J. (1990). Rapid maxillary expansion in the treatment of nocturnal enuresis. *The Angle Orthodontist*.

[2] Kurol J., Modin H., Bjerkhoel A. (1998). Orthodontic maxillary expansion and its effect on nocturnal enuresis. *Angle Orthodontist*.

[3] Usumez S., Işeri H., Orhan M., Basciftci F. A. (2003). Effect of rapid maxillary expansion on nocturnal enuresis. *Angle Orthodontist*.

[4] Schütz-Fransson U., Kurol J. (2008). Rapid maxillary expansion effects on nocturnal enuresis in children. *Angle Orthodontist*.

[5] Fritz G., Rockney R., Bernet W. (2004). Practice parameter for the assessment and treatment of children and adolescents with enuresis. *Journal of the American Academy of Child & Adolescent Psychiatry*.

[6] Butler R. J. (2004). Childhood nocturnal enuresis: developing a conceptual framework. *Clinical Psychology Review*.

[7] Eiberg H., Berendt I., Mohr J. (1995). Assignment of dominant inherited nocturnal enuresis (ENUR1) to chromosome 13q. *Nature Genetics*.

[8] Nevéus T. (2011). Nocturnal enuresis-theoretic background and practical guidelines. *Pediatric Nephrology*.

[9] AbdelFatah D., Shaker H., Ismail M., Ezzat M. (2009). Nocturnal polyuria and nocturnal arginine vasopressin (AVP): a key factor in the pathophysiology of monosymptomatic nocturnal enuresis. *Neurourology and Urodynamics*.

[10] Sinha D., Guilleminault C. (2010). Sleep disordered breathing in children. *Indian Journal of Medical Research*.

[11] Aydil U., Işeri E., Kizil Y., Bodur Ş., Ceylan A., Uslu S. (2008). Obstructive upper airway problems and primary enuresis nocturna relationship in pediatric patients: reciprocal study. *Journal of Otolaryngology—Head and Neck Surgery*.

[12] Gozal D., O'Brien L. M. (2004). Snoring and obstructive sleep apnoea in children: why should we treat?. *Paediatric Respiratory Reviews*.

[13] Xu Z., Cheuk D. K. L., Lee S. L. (2006). Clinical evaluation in predicting childhood obstructive sleep apnea. *Chest*.

[14] Umlauf M. G., Chasens E. R. (2003). Bedwetting—not always what it seems: a sign of sleep-disordered breathing in children. *Journal for Specialists in Pediatric Nursing*.

[15] Alexopoulos E. I., Kaditis A. G., Kostadima E., Gourgoulianis K. (2005). Resolution of nocturnal enuresis in snoring children after treatment with nasal budesonide. *Urology*.

[16] Basha S., Bialowas C., Ende K., Szeremeta W. (2005). Effectiveness of adenotonsillectomy in the resolution of nocturnal enuresis secondary to obstructive sleep apnea. *Laryngoscope*.

[17] Robson W. L. M., Leung A. K. C. (2000). Secondary nocturnal enuresis. *Clinical Pediatrics*.

[18] Lofaso F., Coste A., D'Ortho M. P. (2000). Nasal obstruction as a risk factor for sleep apnoea syndrome. *European Respiratory Journal*.

[19] Pirelli P., Saponara M., Guilleminault C. (2004). Rapid maxillary expansion in children with obstructive sleep apnea syndrome. *Sleep*.

[20] Villa M. P., Malagola C., Pagani J. (2007). Rapid maxillary expansion in children with obstructive sleep apnea syndrome: 12-month follow-up. *Sleep Medicine*.

[21] Compadretti G. C., Tasca I., Bonetti G. A. (2006). Nasal airway measurements in children treated by rapid maxillary expansion. *The American Journal of Rhinology*.

[22] Harvold E. P., Tomer B. S., Vargervik K., Chierici G. (1981). Primate experiments on oral respiration. *American Journal of Orthodontics*.

[23] Weider D. J., Sateia M. J., West R. P. (1991). Nocturnal enuresis in children with upper airway obstruction. *Otolaryngology—Head and Neck Surgery*.

[24] Principato J. J. (1991). Upper airway obstruction and craniofacial morphology. *Otolaryngology—Head and Neck Surgery*.

[25] Barone J. G., Hanson C., DaJusta D. G., Gioia K., England S. J., Schneider D. (2009). Nocturnal enuresis and overweight are associated with obstructive sleep apnea. *Pediatrics*.

[26] Brooks L. J., Topol H. I. (2003). Enuresis in children with sleep apnea. *Journal of Pediatrics*.

[27] Yeung C.-K. (2003). Nocturnal enuresis (bedwetting). *Current Opinion in Urology*.

[28] Çinar U., Vural C., Çakir B., Topuz E., Karaman M. I., Turgut S. (2001). Nocturnal enuresis and upper airway obstruction. *International Journal of Pediatric Otorhinolaryngology*.

[29] Cistulli P. A., Palmisano R. G., Poole M. D. (1998). Treatment of obstructive sleep apnea syndrome by rapid maxillary expansion. *Sleep*.

[30] Pirelli P., Saponara M., Attanasio G. (2005). Obstructive Sleep Apnoea Syndrome (OSAS) and rhino-tubaric disfunction in children: therapeutic effects of RME therapy. *Progress in Orthodontics*.

[31] Villa M. P., Rizzoli A., Miano S., Malagola C. (2011). Efficacy of rapid maxillary expansion in children with obstructive sleep apnea syndrome: 36 months of follow-up. *Sleep and Breathing*.

[32] Rose E., Schessl J. (2006). Orthodontic procedures in the treatment of obstructive sleep apnea in children. *Journal of Orofacial Orthopedics*.

[33] Freeman R. D., Menolascino F. J. (1970). Psychopharmacology and the retarded child. *Psychiatric Approach to Mental Retardation*.

[34] de Bruyne E., van Hoecke E., van Gompel K. (2009). Problem behavior, parental stress and enuresis. *The Journal of Urology*.

[35] Van Tijen N. M., Messer A. P., Namdar Z. (1998). Perceived stress of nocturnal enuresis in childhood. *British Journal of Urology*.

[36] Van Leerdam F. J. M., Blankespoor M. N., Van Der Heijden A. J., Hiraing R. A. (2004). Alarm treatment is successful in children with day- and night-time wetting. *Scandinavian Journal of Urology and Nephrology*.

[37] Gozmen S., Keskin S., Akil I. (2008). Enuresis nocturna and sleep quality. *Pediatric Nephrology*.

[38] Nevéus T., Läckgren G., Tuvemo T., Hetta J., Hjälmås K., Stenberg A. (2000). Enuresis—background and treatment. *Scandinavian Journal of Urology and Nephrology, Supplement*.

[39] Hjälmås K., Arnold T., Bower W. (2004). Nocturnal enuresis: an international evidence based management strategy. *Journal of Urology*.

